# Are there sex differences in spatial reference memory in the Morris water maze? A large-sample experimental study

**DOI:** 10.3758/s13420-023-00598-w

**Published:** 2023-09-18

**Authors:** Candela Zorzo, Jorge L. Arias, Marta Méndez

**Affiliations:** 1 Department of Psychology, University of Oviedo, Faculty of Psychology, Plaza Feijoo s/n, 33003 Oviedo, Asturias, Spain; 2 Instituo de Neurociencias del Principado de Asturias (INEUROPA), Faculty of Psychology, Plaza Feijoo s/n, 33003 Oviedo, Asturias, Spain; 3 Instituto de Investigación Sanitaria del Principado de Asturias (ISPA), Av. del Hospital Universitario, s/n, 33011 Oviedo, Asturias, Spain

**Keywords:** Morris water maze, Spatial navigation, Learning, Sex differences, Females

## Abstract

Sex differences have been found in allocentric spatial learning and memory tasks, with the literature indicating that males outperform females, although this issue is still controversial. This study aimed to explore the behavior of male and female rats during the habituation and learning of a spatial memory task performed in the Morris Water Maze (MWM). The study included a large sample of 89 males and 85 females. We found that females searched slightly faster than males during habituation with a visible platform. During learning, both male and female rats decreased the latency and distance traveled to find the hidden platform over the days, with males outperforming females in the distance traveled. Females swam faster but did not find the platform earlier, suggesting a less directed navigational strategy. Both sexes increased time spent in the target zone over the days, with no sex differences. Although females swam more in the periphery during the first days of the task, both sexes decreased the time spent in this area. Finally, only males increased swimming in the pool's center over the days, spending more time than females in this area across the entire training. In conclusion, we need to register several variables in the MWM and analyze path strategies to obtain more robust results concerning sex differences. Research on spatial learning should include both sexes to achieve a more equitable, representative, and translational science.

## Introduction

Memory can be defined as the ability to classify, encode, store, and recover previously learned information (Asok et al., 2019; Paul et al., 2009). When this information has a spatial component and allows us to reach a goal location and situate ourselves in the surrounding environment, it is known as *spatial memory*, which requires spatial navigation (Wolbers & Hegarty, 2010). This ability is an indispensable cognitive function that needs the interaction of multiple cognitive processes, from the perception of sensorial and proprioceptive stimuli to storage and later retrieval (Chersi & Burgess, 2015).

For successful navigation, subjects – both humans and animals – depend on two strategies or frames of reference, which can be alternated and combined (Colombo et al., 2017). These are the allocentric and egocentric strategies. The allocentric strategy refers to spatial orientation using visual distal cues, that is, the ability to learn and remember the location of certain environmental reference points and to establish a spatial relationship between them, known as *cognitive mapping* (Epstein et al., 2017; Tolman, 1948). In contrast, the egocentric strategy depends on signals about the position and movement of the organism (vestibular, proprioceptive, and motor information) that may be updated during locomotion and do not need external cues (Burgess, 2008).

Sex differences in spatial cognition have received substantial attention and are still a topic of considerable debate. Some reviews and meta-analyses reflect that males usually perform better than females in some spatial abilities, such as mental rotation, spatial working memory, and spatial orientation, whereas the opposite is found in location memory (Cimadevilla & Piccardi, 2020; Jonasson, 2005; Linn & Petersen, 1985; Voyer et al., 2017; Yagi & Galea, 2019). Particularly in spatial navigation, compelling theories support that males’ advantage comes from an evolutionary perspective, considering the idea that males rely on spatial-navigational skills more than females to achieve demanding survival practices (Chen et al., 2020; Levine et al., 2016). Sex differences are often observed in humans (Castillo et al., 2021; Fernandez-Baizan et al., 2019; León et al., 2016; Sneider et al., 2015; Yu et al., 2021), although a recent meta-analysis showed a small to moderate effect (Nazareth et al., 2019). Some variables linked to sex differences are pointed out, such as a differential processing of spatial information and stimuli perception (Herrera et al., 2019), the strategy employed (Pletzer et al., 2019), task level difficulty (Chamizo et al., 2020; Chen et al., 2020; Tascón et al., 2021), familiarity with the environment (De Goede & Postma, 2015; Tascón et al., 2021), familiarity with tasks that depend on visuospatial abilities (Rodriguez-Andres et al., 2018), or anxiety (Munoz-Montoya et al., 2019). Also, it is important to note that the study performed by Coutrot et al. (2018), using a global sample, determined that spatial navigational differences are removed when cultural, level of equality, and wealth are considered.

Rodent studies align with human assessments, as males usually outperform females in the Morris Water Maze (MWM) task (Mifflin et al., 2021; Qi et al., 2016; Safari et al., 2021; Simpson & Kelly, 2012; Woolley et al., 2010; Yagi et al., 2017). Interestingly, some authors attribute better male performance to different strategy choices (Duarte-Guterman et al., 2015; Shansky, 2018), as well as differential attention to specific landmark features (Chamizo et al., 2014) or swim patterns (Devan et al., 2016). Recent articles indicate that sex differences could even rely on the motivation to complete the task, as well as the researcher’s manipulation, revealing a male advantage in the MWM, but a female outperformance in the IntelliCage, where animals are maintained in their social environment (Mifflin et al., 2021).

Overall, sex differences in spatial cognition are still a controversial issue, as many studies show no differences, either in humans or rodents (Bucci et al., 1995; Devan et al., 2016; Macúchová et al., 2017; Munoz-Montoya et al., 2019; Zorzo et al., 2020). The review of Jonasson (2005) reported no differences between male and female rats in half of the studies (noted by Blokland et al., 2006). According to Faraji et al. (2010), some of the differences are not shown consistently, suggesting that many of them may be responding to sex hormones, but also to parameters that depend on the task. Contradictory results are likely to arise due to methodological and statistical factors. Thus, an important limitation when assessing sex differences might be the sample size of the groups (Voyer et al., 2007).

Spatial memory in rodent models can be assessed through distinct behavioral tasks. However, given its multiple advantages, the MWM has been one of the most used by the scientific community (Vorhees & Williams, 2014a). Furthermore, since the advent of computer-based virtual environments, adaptations of the MWM – virtual MWM – are used to evaluate spatial memory in humans (Astur et al., 1998; Ferguson et al., 2019; Kuhn et al., 2018; Schoenfeld et al., 2017).

Considering all the above, we aimed to behaviorally explore sex differences in spatial learning and memory using the MWM, and including a wide range of behavioral parameters in a large sample size of 85 female and 89 male rats.

## Material and methods

### Subjects

To carry out these experiments, a total of 89 male and 85 female 10- to 12-week-old Wistar rats were employed from the Production Center and Animal Experimentation of the University of Seville, Spain. All animals were housed in groups of four subjects per cage (38 × 55 × 20 cm) with ad libitum food and water availability. They were housed in an environment with standard ventilation conditions, a constant temperature of 22 ± 2° C, a relative humidity of 65–75%, and a light-dark cycle of 12 h (light: 08:00–20:00 h; darkness: 20:00–08:00 h).

All procedures and handling of animals were carried out following the European Directive 2010/63/EU and Royal Decree 53/2013 (BOE-A-2013-1337) of the Government of Spain, and were approved by the local committee for animal studies at Oviedo University. According to the legislation and guidelines governing the ethics of animal use, we consider it important to highlight that all the subjects employed in this research were previously used for other research aims. In this research, we collected new data, analyzing parts of the protocol that were identical across experiments. The experience that animals had was identical up to the final of the MWM learning. Thus, our aim was to provide useful data to the research community.

### Experimental procedure

Prior to the behavioral tests, the animals were handled daily for 1 week to reduce the stress generated by contact with the experimenter. In the case of females, vaginal smears we collected, performing a direct cytology for three consecutive days 1 week before the learning procedure. With this procedure, we wanted to verify females have cyclical fluctuations. All the rats showed a normal estrous cycle. These procedures were performed between 8:00 and 10:00 h.

As for the spatial memory procedure, male and female rats were habituated to the training in the MWM pool for 1 day to avoid the stress caused by contact with the experimenter and the contingencies of the task. Then, animals were trained for five consecutive days on an allocentric spatial reference memory task performed in the MWM. Training was conducted with five visual cues with different volumes and color patterns surrounding the pool. Behavioral tests were performed between 10:00 and 13:00 h.

### Behavioral procedure

#### Apparatus

**Fig. 1:**
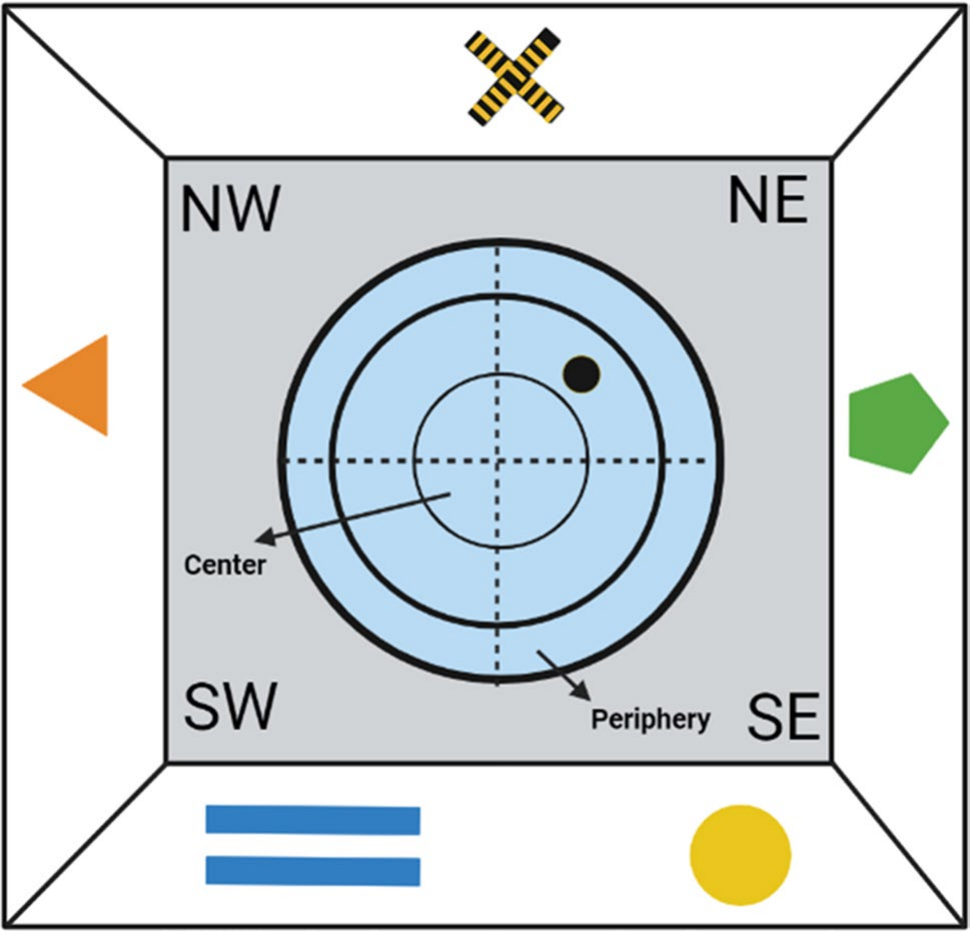
Schematic representation of the cue arrangement and pool design

Allocentric spatial learning was evaluated in the MWM (Morris, 1984). The pool consisted of a black circular fiberglass tank measuring 150 cm in diameter and 40 cm high, and was filled with tap water at a temperature of 22 ± 2° C. Inside the MWM, there was a hidden escape platform 2 cm beneath the water’s surface, 10 cm in diameter and 28 cm in height. The pool was divided into four imaginary quadrants (NE, NW, SE, SW) to locate the start positions, and the escape platform was located in the center of quadrant NE. The MWM was surrounded by black panels located 50 cm away from the maze, on which we placed five distal visual cues. The cues were selected with different colors and shapes: a green pentagon, an orange triangle, two horizontal blue bars, a yellow circle, and a cross in yellow and black. The five cues were identical, and located in the same place across all days, for all groups (Fig. 1). The MWM was located in the center of a 16 m^2^ room illuminated by an indirect 4,000 lx light from two lamps facing the walls of the room.

The animal’s behavior was recorded with a video camera (V88E, *Sony, Spain*) located above the pool, and using a computerized video-tracking system (Ethovision XT 14.0, Noldus Information Technologies, Wageningen, The Netherlands).

#### Habituation

The first day of the protocol was devoted to habituation to the testing contingencies of the behavioral phase. Male and female rats were subjected to four trials in which they had to reach a visible white platform that protruded 2 cm from the water and was located in the center of the pool. On each trial, the subjects were released from each quadrant (NE, NW, SE, SW) facing the pool wall, following a pseudo-randomized sequence. The trial duration lasted for 60 s, and once the animals found the platform, they were maintained there for 15 s. The inter-trial interval (ITI) was 30 s. If animals failed to reach the platform, they were guided and maintained there for 15 s. Once the habituation session had ended, the animals were carefully dried and returned to their home cage.

We recorded latencies to reach the visible platform.

#### Learning phase

On the following five days after habituation, rats were required to locate a hidden platform placed in the center of the target quadrant (NE). Training was performed in blocks of six trials per day with a fixed ITI of 30 s, during which the animals were placed in a black bucket. Trials consisted of four acquisition trials, one learning probe trial, and one additional trial to avoid possible learning extinction. In the acquisition trials, rats had to reach the hidden platform. Once the rats had found the platform, they remained in the reinforced place for 15 s. If the animals failed to reach the platform after 60 s, they were guided and placed on it for 15 s. On each trial, the subjects were released from each quadrant (NE, NW, SE, SW) facing the pool wall, following a pseudo-randomized sequence that varied during the five days of the learning task, but that was the same for all the male and female subjects. The learning probe trial consisted of a 60-s trial in which the escape platform was removed, and the rat was introduced from the opposite quadrant (SW) to where the platform had been in previous trials. If the animals failed to reach the platform after 60 s, they were gently guided to the platform and were maintained there for 15 s. Finally, rats received an additional trial with the platform in its usual position to avoid possible learning extinction. This trial was also pseudo-randomized across the five days, and was the same for all animals. Once the training session had ended, the animals were carefully dried and returned to their home cage.

We recorded latencies, distance travelled, and swimming speed in the acquisition trials, time spent in NE quadrant (target) in the probe trial, and time spent in the periphery and center of the pool (see Fig. 1) in the probe trial.

### Statistical analysis

Mean latency of the habituation day was compared between groups using a Mann-Whitney Rank Sum Test. The escape latencies (s), distance travelled (cm), and swimming speed (cm/s) during the learning phase were analyzed using a 2 × 5 mixed ANOVA (inter-group factor: sex, two levels; intra-group factor: days, five levels). Latencies, distance, and speed for the four acquisition trials were averaged per day. The permanency in the target quadrant (NE) during the probe trial was analyzed by comparing the time spent, using a 2 × 5 mixed ANOVA (inter-group factor: sex, two levels; intra-group factor: days, five levels). Time spent in the periphery during the probe test was analyzed by comparing the time spent, using a 2 × 5 mixed ANOVA (inter-group factor: sex, two levels; intra-group factor: days, five levels). Same analysis was performed to evaluate time spent in the center of the pool, as well as swimming speed. Sex differences in latency of trial one in the second, third, fourth, and fifth days was analyzed by comparing latencies, using a 2 × 5 mixed ANOVA (inter-group factor: sex, two levels; intra-group factor: trials, four levels). When an interaction effect was found, we performed post hoc multiple comparisons considering the interaction of two factors. When the interaction effect was not found, but there were differences in the main effects, we performed post hoc analysis considering the significant factors. For all multiple comparisons, we employed the Holm-Sidak method. Power (1 – β) analysis was performed with alpha 0.05, and is described when significant differences were found.

All the data were analyzed with the SigmaStat 14 program (Systat, Richmond, CA, USA) and expressed as means ± standard error of mean (SEM). Statistical significance was set at the .05 level. For graphic representation, we employed the SigmaPlot 14 program (Systat).

### Habituation trials

**Fig. 2:**
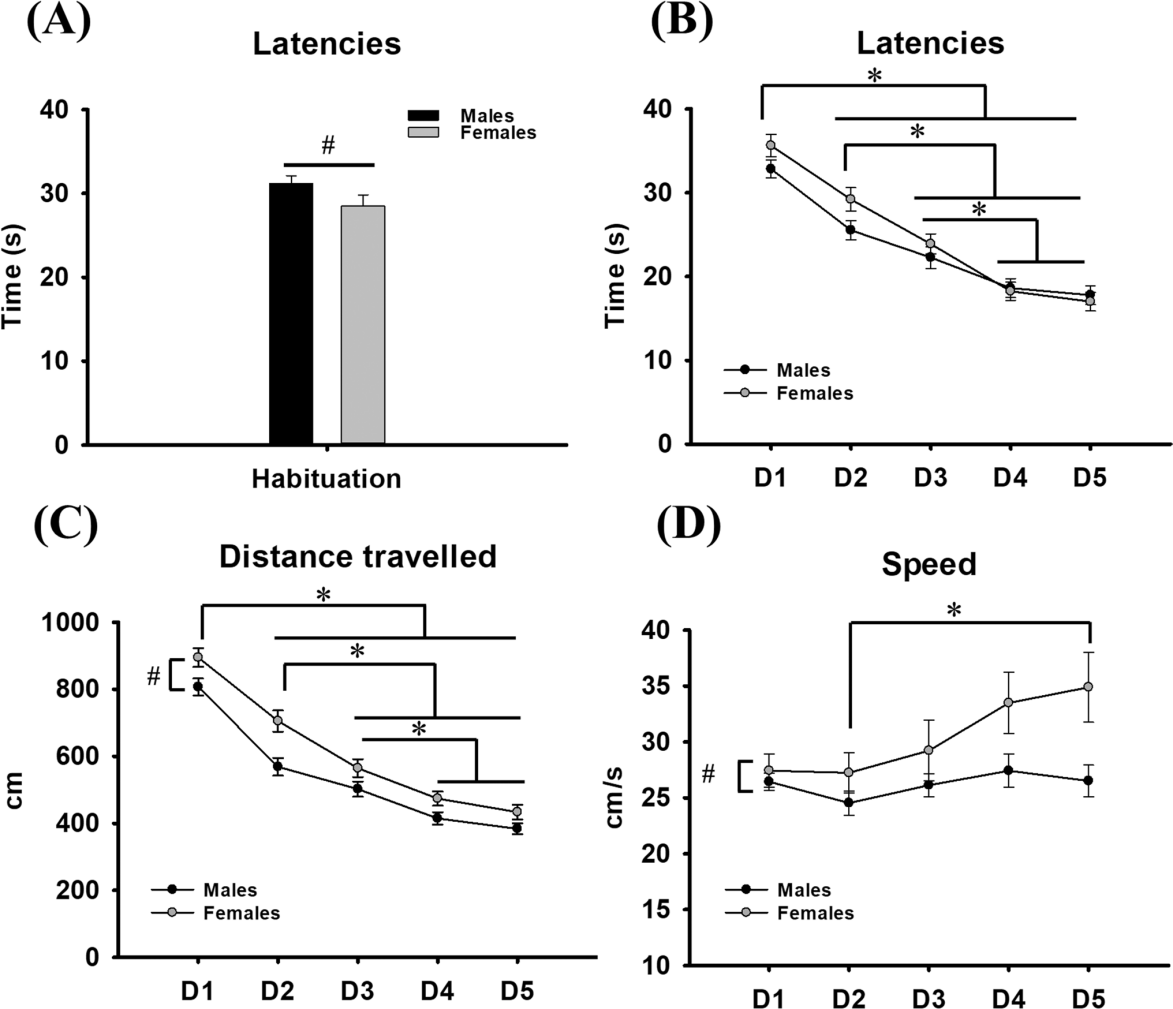
(**A**) Male and female latencies in reaching the platform during the habituation. (**B**) Male and female latencies in reaching the platform during the learning task. (**C**) Male and female distances travelled to reach the platform during the learning task. (**D**) Male and female swimming speeds to reach the platform during the learning task. * represents differences across days for both sexes. # represents sex differences. Data are expressed as mean ± SEM

On the habituation day, females reached the visible platform before the males (*U* = 2,980, n_1_ = 89; n_2_ = 85; *P* = 0.016) (Fig. 2A).

### Acquisition trials: Latency, distance, and speed

Regarding latencies, there was no interaction with sex × day (*F*_(4, 688)_ = 1.723; *P* = 0.143), nor sex differences (*F*_(1, 172)_ = 1.719; *P* = 0.192). However, latencies to reach the platform showed differences between days (*F*_(4, 688)_ = 87.620; *P* < 0.001; *β =* 1.000). We performed multiple comparisons across the factor day, and it was revealed that both sexes showed longer latencies to reach the platform on day one compared to the rest of days (*P* < 0.001), on day two compared to days three, four, and five (*P* < 0.001), and on day three compared to days four and five (*P* < 0.001) (Fig. 2B).

As for distance travelled during the learning phase, there were no interaction effect (*F*_(4, 688)_ = 1.335; *P* = 0.255), whereas differences were found between sexes (*F*_(1, 172)_ = 14.693; *P* < 0.001; *β =* 0.972) and between days (*F*_(4, 688)_ = 137.458; *P* < 0.001; *β =* 1). We applied the Holm-Sidak method to decipher differences in the main factors, and it was revealed that females showed more distance travelled to reach the platform compared to males (*P* < 0.001). Moreover, both sexes presented a higher distance travelled on day one compared to the rest of the days (*P* < 0.01), on day two compared to days three, four, and five (*P* < 0.01), and on day three compared to days four and five (*P* < 0.01) (Fig. 2C).

Speed analysis showed no interaction with sex × day (*F*_(4, 688)_ = 1.582; *P* = 0.177), whereas there were differences between sexes (*F*_(1, 172)_ = 6.329; *P*= 0.013; *β* = 0.631) and between days (*F*_(4, 688)_ = 3.379; *P* = 0.009; *β =* 0.687). Post hoc analysis in the main factors revealed that females swam faster than males (*P* = 0.013). Speed across days revealed an increased velocity on day five, compared to the second day of the task (*P* = 0.037) (Fig. 2D).

### Probe trials: Percentage of time spent in the target quadrant (NE)

**Fig. 3:**
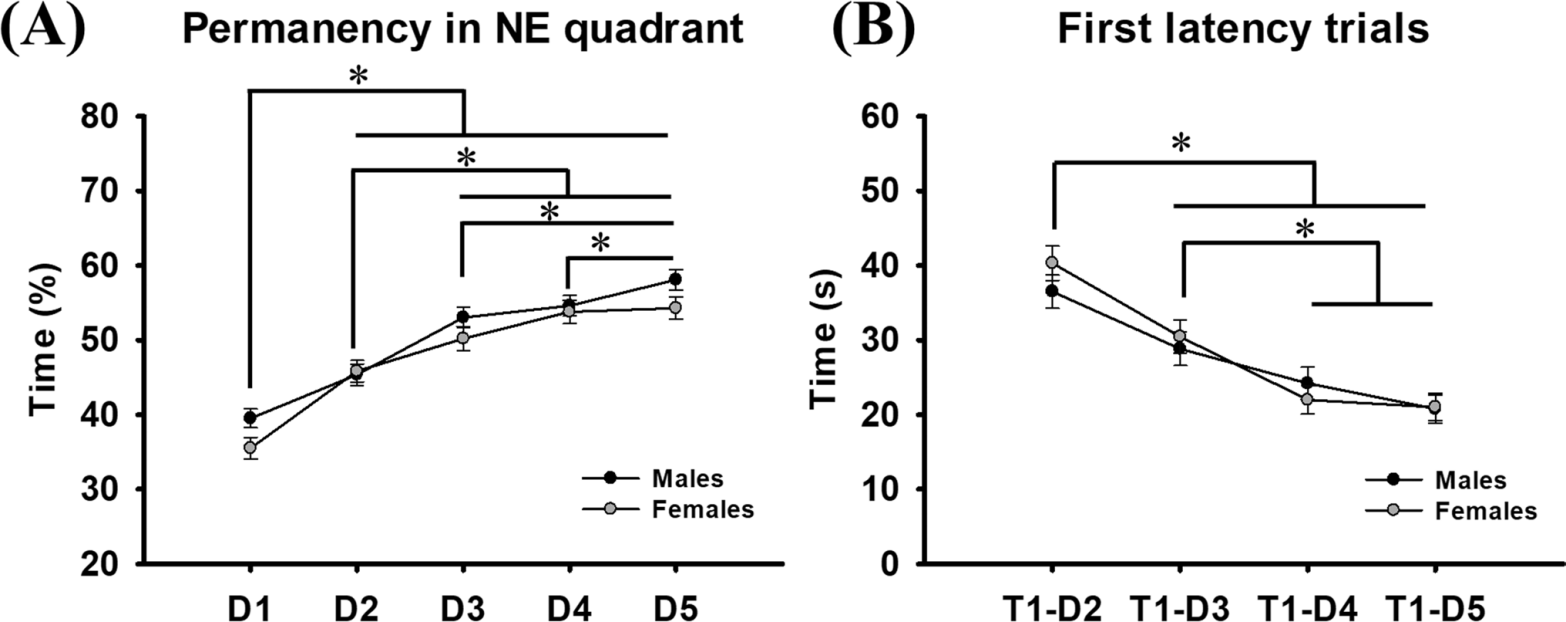
(**A**) Male and female time spent in the target quadrant. * represents differences across days for both sexes. (**B**) Male and female latencies in reaching the platform during the first trial each day. Data are expressed as mean ± SEM

The analysis of time spent in the target quadrant revealed there was not a sex × day effect (*F*_(4, 688)_ = 1.260; *P* = 0.285), nor sex differences (*F*_(1, 172)_ = 2.835; *P* = 0.094), whereas day differences were found (*F*_(4, 688)_ = 75.955; *P* < 0.001; *β* = 1.000). Post hoc analysis showed there was an increase in time spent in the target zone across days. Subjects showed less time swimming in the target zone during the first day compared to the rest of days (*P* < 0.001), on the second day compared to days three, four, and five (*P* < 0.001), and on the third and fourth days compared to fifth (*P* < 0.001) (Fig. 3A).

### Acquisition trials: Latency in the fist trial of days two, three, four, and five

The analysis of time spent to reach the platform in the first trail each day revealed there was not a sex × day effect (*F*_(3, 516)_ = 0.883; *P* = 0.450), nor sex differences (*F*_(1, 172)_ = 0.213; *P* = 0.645), whereas differences were found on trial one across days of training (*F*_(3, 516)_ = 34.131; *P* < 0.001; *β* = 1.000). Post hoc analysis showed there was a reduction in time to reach the platform in the first trial across days. Subjects spent more time to reach the platform on day two compared with days three, four, and five (*P* < 0.001), and on the third day compared to days four and five (*P* < 0.001) (Fig. 3B).

### Probe trials: Percentage of time spent in the periphery and center

**Fig. 4:**
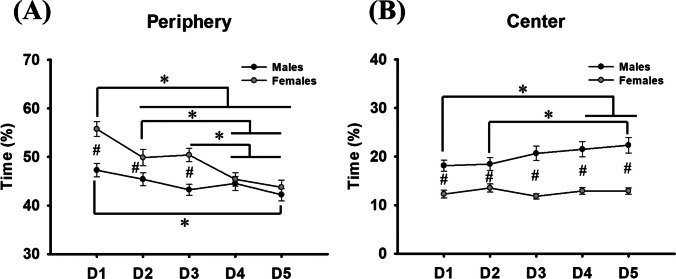
(**A**) Male and female time spent in the periphery of the pool. (**B**) Male and female time spent in the center of the pool. * represents differences across days for males and/or females. # represents sex differences. Data are expressed as mean ± SEM

Regarding time spent in different zones of the maze, analysis of swimming on the periphery showed an interaction effect sex × day (*F*_(4, 688)_ = 3.785; *P* = 0.005; *β =* 0.768). Differences were found between sexes (*F*_(1, 172)_ = 12.995; *P* < 0.001; *β =* 0.949) and between days (*F*_(4, 688)_ = 13.693; *P* < 0.001; *β =* 1.000). Multiple comparisons revealed that female rats presented higher swimming time on the periphery on days one (*P* < 0.001), two (*P* = 0.025), and three (*P* < 0.001). Also, the Holm-Sidak method showed that the male group spent less time in the periphery on the last day compared with the first day (*P* < 0.001). Female rats spent more time in the periphery of the pool on the first day compared to days two (*P* = 0.005), three (*P* = 0.012), four, and five (*P* < 0.001), more time on the second day compared to days four (*P* = 0.031), and five (*P* = 0.003), and more time on the third day compared to days four (*P* = 0.016) and five (*P* = 0.001) (Fig. 4A).

Regarding the center of the MWM, analysis showed an interaction effect sex × day (*F*_(4, 688)_ = 2.962; *P* = 0.019; *β =* 0.587). Differences were found between sexes (*F*_(1, 172)_ = 33.816; *P* < 0.001; *β =* 1.000) and between days (*F*_(4, 688)_ = 3.029; *P* = 0.017; *β =* 0.603). Post hoc analysis showed differences between sexes on all days of the task, where the male group swam for more time in this area (day 1: *P* < 0.001; day 2: *P* = 0.003; day 3: *P* < 0.001; day 4: *P* < 0.001; day 5: *P* < 0.001). Also, the male group swam for more time in the center on the last two days of the task, compared to the first day (day 4: *P* = 0.024; day 5: *P* = 0.002), and on the last day compared to the second (*P* < 0.006). Females did not show differences across days (*P* > 0.05) (Fig. 4B).

## Discussion

The aim of the present study was to explore the behavior of male and female rats during the acquisition of a spatial memory task assessed through MWM. Females showed a slightly faster search time than males during habituation with a visible platform. During the learning phase, male and female rats decreased the latency and distance travelled in finding the hidden platform. A similar decrease was observed in the first trial from days two to five. No sex differences were found in latency, but males travelled less distance to reach the platform. Regarding swimming speed, only females increased speed while training, and this group presented higher speed than males. Both male and female rats increased the time spent in the target zone of the MWM, with no sex differences. Both males and females decreased the time spent in the periphery (thigmotaxis). Females showed a greater swimming time in the periphery compared to males during the first three days of the task, thereafter males and females showed similar periphery swimming. Finally, only males increased swimming in the center of the pool over the days, spending more time in this area in comparison with females across all the training.

Habituation in the MWM is usually performed to promote adaptation to the test environment and reduce the stress that could be caused by the task contingencies (Belviranli et al., 2012; Marcotte et al., 2021; Nategh et al., 2015; Vorhees & Williams, 2014b). Thus, the purpose is to allow animals to become familiar with water temperature, swimming, reaching the platform, and the researcher’s manipulation. This habituation is common and necessary in other behavioral tasks that measure memory (Ali et al., 2017; Dawood et al., 2020; McCormick et al., 2010; Méndez-López et al., 2009) as well as anxious behavior (Sorregotti et al., 2018) or other procedures that assess locomotor activity, exploration, or motor features (Bert et al., 2002; Jacquez et al., 2021; Poveda et al., 2020). This indicates the relevance of habituating animals to prevent confounding results that may not be linked to the task.

Here, we have found sex differences in habituation, revealing that female rats reached the platform faster than males. These results reflect the need to include habituation in spatial memory tasks, which is commonly performed in a similar way to this study (Anderson et al., 2013; Arias et al., 2012; Conejo et al., 2010), only to swimming activity (Bert et al., 2002; Nategh et al., 2015) or to platform exploration (Maehata et al., 2020). Regarding habituation analysis, we can hypothesize that the swimming speed was higher in females not only during the acquisition trials, but also during the habituation. Simpson and Kelly (2012) propose in their review that females display greater baseline activity levels than males. In addition, it has been reported that during habituation or the first days of training, both female rats and women may feel higher levels of anxiety, in some cases, associated with faster swimming, as well as thigmotaxis (Coluccia & Louse, 2004; Simpson & Kelly, 2012; Treit & Fundytus, 1988). Our speed results during learning are in line with this notion, as females swam faster than their counterparts. Also, females show thigmotaxic behavior during the first days of training (discussed below), supporting this hypothesis. If we observed this behavior during the acquisition trials, a similar speed and peripheral results during habituation could have been expected (not recorded).

We trained the animals for five consecutive days, with four trials a day in which rats had to reach the nonvisible platform, one 60-s probe trial each day without the platform to assess memory performance, and one additional trial with the platform to prevent learning extinction. Overall, both the male and female groups improved their performance in reaching the platform, as a progressive reduction of latencies across days could be observed. These results are supported by the distance travelled, which decreased over days in the same manner as latencies, reflecting that the performance of animals progresses over training, as is commonly reported (Belviranli et al., 2012; Chamizo et al., 2016; Faraji et al., 2010; Macúchová et al., 2017; Mazor et al., 2009; Mifflin et al., 2021). In addition, we performed an analysis to assess memory retention with a 24-h interval. Thus, we compared male-female performance in reaching the platform in the first trial on days two, three, four, and five, and we observed a reduction on latency across days of training suggesting a conserved 24-h memory retention, as well as analogous performance between sexes, comparable to others who observed similar distances swam in the first trial of the second, third (Mancini et al., 2021), and last days of the task (Chow et al., 2013)

Regarding sex differences, we did not detect any among latencies, similar to some previous studies (Faraji et al., 2010; Mazor et al., 2009; Qi et al., 2016), but contrary to others (Chow et al., 2013; Mifflin et al., 2021; Safari et al., 2021). However, sex differences were found in total distance travelled. Males displayed shorter swimming paths to reach the submerged platform, revealing a more efficient performance than female rats, similar to other reports (Anderson et al., 2013; Chow et al., 2013; Safari et al., 2021). Taking into consideration speed analysis, we observed that female rats increase swimming velocity from day two to the last day of the task, while male rats remained constant. Additionally, as described above, females showed a greater swimming speed than males. These results are in line with the study of Tucker et al. (2016) and – when interpreted in combination with latency and distance – it is possible to propose an advantage of males during the learning acquisition. The two sexes reached the platform in comparable time, but females swim faster and travelled longer distances, suggesting a less directed path before reaching the platform.

Another common manner for assessing learning acquisition is the exploration of time spent in the target part of the pool. Usually, the MWM is imaginary, divided into four quadrants, and the submerged platform remains in one of them. During the probe trial, the platform is removed, allowing testing of whether animals have learned its location. Here, we have confirmed the success in learning, as both male and female rats increased the time spent in the target zone of the MWM, suggesting a consolidation of memory information. The percentage of time for males and females increased almost 20% (males: 18.57%; females: 18.78%) from day one to day five, showing higher rates from day one of training. Specifically, the percentage spent in the NE quadrant in males on day one is 39.51% (SEM: 1.24) and on day five is 58.08% (SEM: 1.39). Females spent 35.05% (SEM: 1.44) on day one, and 54.28% (SEM: 1.50) on the last day of training. These percentages are commonly reported from the beginning of training and reflect a good and progressive learning on the allocentric spatial task (Banqueri et al., 2017; Gutiérrez-Menéndez et al., 2019). Nevertheless, we did not detect sex differences in the probe trial, similar to Faraji et al. (2010) and Qi et al. (2016). Along this line, some rodent studies observed that both sexes succeed in the spatial task (Belviranli et al., 2012; Chamizo et al., 2016; Mazor et al., 2009), which was also found in humans assessed with virtual MWM, where differences were observed in the escape latencies but not in probe trials (Piber et al., 2018). It may be interesting to study how male and female rats distribute their swimming time during the probe trial in segments of 30 s, which is a limitation of the current study.

It is common to find sex differences (usually in favor of male rodents or men) in spatial memory (Fernandez-Baizan et al., 2019; Mifflin et al., 2021; Qi et al., 2016; Safari et al., 2021; Simpson & Kelly, 2012; Woolley et al., 2010; Yagi et al., 2017), although other studies report comparable male-female responses during learning (Chamizo et al., 2016; Gutiérrez-Menéndez et al., 2019; Mazor et al., 2009; Sebastian et al., 2013). Some authors state that sex differences can be attributed to mental rotation, needed for the acquisition of orthogonal directions (Linn & Petersen, 1985). In fact, there is much evidence about the different strategies that males and females – both humans and animals – use when navigating. Whereas males tend to use geometry as a source of information, females tend to rely on landmarks (Herrera et al., 2019). Thus, although not recorded in this study, it is important to realize that males and females differ in the strategy they use to solve a spatial navigational task (Aguilar-Latorre et al., 2022; Andersen et al., 2012; Chamizo et al., 2016). Moreover, in female rats, it has been found that prior spatial experience leads to a more accurate response, and when compared with males, the sex differences disappeared when the rats had previously dealt with other spatial tasks. However, a more accurate response was observed in males when no prior experiences were allowed (Aguilar-Latorre et al., 2022). The meta-analysis by Jonasson (2005) outlined that training protocols tend to reduce sex differences, which could explain the results of the present study. Thus, we note the relevance of prior habituation or non-spatial experience (Aguilar-Latorre et al., 2022; Perrot-sinal et al., 1996), and consider that the habituation carried out may have reduced the sex differences in the latency and time spent in the objective quadrant.

Furthermore, it has been indicated that females tend to encode, store, and recover detailed peripheral information, whereas males usually code and recall information central to the event (Herrera et al., 2019). Interestingly, Chamizo et al. (2016) observed sex differences after environmental enrichment in rats that performed a spatial navigational task, suggesting that environmental enrichment leads to a reduced anxiety response measured by thigmotaxis while swimming in the pool. Also, it was revealed that male and female rats differed in the strategy employed, indicating that females prefer landmark cues instead of using information about pool geometry (Chamizo et al., 2016). In this article, although differences were found in the strategy employed, both sex groups performed the task successfully, similar to our findings. When tested individually, both sexes can use both sources of information to reach the platform, but a clear advantage for males in using geometrical information was found (Chamizo et al., 2016).

One way to approach the study of path strategy can be reflected in the analysis of the time spent in each part of the pool, at least indirectly. We observed that both males and females decreased the time spent in the periphery across task days, with a higher decrease observed in females. Males decreased from day one to day five, meanwhile females progressively reduced their peripheral swimming. As a result, during the first three days of the task it is possible to observe higher peripheral swimming in females, as others have previously reported (Harris et al., 2008; Perrot-Sinal et al., 1996), but the difference disappears on the last two days of the task, when animals are well familiarized with the contingency of the task. Peripheral swimming results were complemented with the center measures, where males increased over days, whereas females showed no differences. Moreover, it was observed that males spent more time in the center compared with females during the five consecutive days. Thus, it can be hypothesized that throughout training, males develop a navigational strategy that varies across days, with a slight decrease of searching in the periphery and an increase in swimming across the center. As for females, some studies show they are more apt to explore with thigmotaxis (Devan et al., 2016), suggesting a female preference for approaching the wall, a local cue. As increased peripheral swimming can be linked to a general increase in stress or anxiety, it can be considered the opposite of center swimming, as it usually is interpreted in the open field (Al-Hasani et al., 2015; Tanda et al., 2009). Therefore, we can assume that male rats show less anxious swimming behavior, performing more exploration of the pool’s center, which could be responsible for the slight differences previously reported.

Regarding factors that influence female behavior, a role of hormones (estrogen and progesterone) during the estrous cycle has been claimed, which fluctuate across the four-day rat estrous cycle (Simpson & Kelly, 2012). Poorer spatial reference memory has been found in the proestrous stage than in the estrous cycle phase (Duarte-Guterman et al., 2015; Simpson & Kelly, 2012), although other studies have observed that the estrus and proestrus stages did not show any impact on the learning performance of rats (Berry et al., 1997; Farhadinasab et al., 2009). To our knowledge, estradiol in the hippocampus regulates gene transcription linked to memory consolidation (Bean et al., 2014), and it has been shown that females’ high levels of estradiol may disrupt memory, whereas low levels could facilitate it (Holmes et al., 2002). This suggests that spatial memory, which depends on the hippocampus (Clark et al., 2007), is modulated by estradiol (Barker et al., 2009). We assessed the estrous cycle of female rats only in order to confirm a regular cycle, but we did not associate it with learning, which may be a limitation of this study. However, due to the large number of female rats employed in this study, we assume that the four phases (diestrous, estrous, proestrous, and metaestrous) of the cycle are represented – albeit not differentiated – in the female sample.

In conclusion, we found a comparable behavioral performance of male and female rats in some variables measured – latency to reach the platform and permanencies in the target zone, but an outperformance of males in distance travelled. Also, females swam faster but did not find the platform earlier, suggesting a less directed navigational strategy. The differences in allocentric spatial navigation can be supported by the differences in swimming across the center or the periphery of the pool, proposing an enhanced thigmotaxic behavior of females, limited to the first days of training. Thus, it is important to include the different measures – those included here and others – that can be registered in the MWM, to obtain more robust results.

Finally, this is a large-sample study that provides stronger and more reliable results, allowing us to control false-negative or false-positive findings (Biau et al., 2008). This study highlights the importance of including females in behavioral analyses, as we can find differences in allocentric spatial navigation. Studying both sexes is a requirement that should be taken into account in scientific articles, and it is important to consider that preclinical neuroscience research has conventionally been performed in males, leading to some misinterpretations in females. The inclusion of females leads to a more equitable, representative, and translational science (Shansky & Murphy, 2021).

## Data Availability

The datasets generated and/or analysed during the current study are available from the corresponding author on reasonable request.
